# Spinal Manual Therapy for Adolescent Idiopathic Scoliosis: A Systematic Review and Meta-Analysis of Randomized Controlled Trials

**DOI:** 10.1155/2023/7928429

**Published:** 2023-01-04

**Authors:** Yan Sun, Yong Zhang, Haoning Ma, Mingsheng Tan, Zhihai Zhang

**Affiliations:** ^1^Guang'an Men Hospital, China Academy of Chinese Medical Sciences, No. 5 Beixiange St., Xicheng District, Beijing 100053, China; ^2^Department of Orthopaedics, China-Japan Friendship Hospital, Beijing 100029, China

## Abstract

**Objective:**

We conducted this meta-analysis to provide better evidence of the efficacy of manual therapy (MT) on adolescent idiopathic scoliosis (AIS).

**Methods:**

All RCTs of MT for the management of patients with AIS were included in the present study. The treatment difference between the experimental and control group was mainly MT. The outcomes consisted of the total effective rate, the Cobb angle, and Scoliosis Research Society-22 (SRS-22) questionnaire score. Electronic database searches were conducted from database inception to July 2022, including the Cochrane Library, PubMed, Web of Science, Embase, Wanfang Data, CNKI, and VIP. The pooled data were analyzed using RevMan 5.4 software.

**Results:**

Four RCTs with 213 patients in the experimental group were finally included. There are 2 studies of standalone MT in the experimental group and 3 studies of MT with identical conservative treatments in the control group. Three trials reported total effective rate, and a statistically significant difference was found (*P* = 0.004). Three trials reported Cobb angle, and a statistical difference was found (*P* = 0.01). Then, sensitivity analysis showed that there was a significant difference in the additional MT subgroup (*P* < 0.00001) while not in the standalone MT subgroup (*P* = 0.41). Three trials reported SRS-22 scores (*P* = 0.55) without significant differences.

**Conclusion:**

There is insufficient data to determine the effectiveness of spinal manipulation limited by the very low quality of included studies. High-quality studies with appropriate design and follow-up periods are warranted to determine if MT may be beneficial as an adjunct therapy for AIS. Currently, there is no evidence to support spinal manipulation.

## 1. Introduction

Adolescent idiopathic scoliosis (AIS) is a complex three-dimensional deformity in which one or more segments of the spine bend laterally with rotation of the vertebral body occurring at or around puberty [1]. AIS accounts for approximately 80% of confirmed scoliosis cases [2]. Moreover, it is also the most rapid progress that coincides with the adolescent growth spurt, which needs to be adequately managed. At present, the underlying pathogenesis has not been fully elucidated, which may be closely correlated with factors such as genetic components, bone dysplasia, endocrine dysfunction, and acquired undesirable posture. Severe AIS may lead to abnormalities including a shaver back, lopsided shoulder, and thoracic malformation and even affect the cardiopulmonary function and irreversible damage to nerves. Therefore, early diagnosis, prevention, and treatment were essential for AIS [3]. Owing to the physical and psychological trauma, and the inevitable negative impact of internal fixation on growth and development, surgical treatment was widely accepted as an alternative treatment for patients with Cobb angle of more than 40°, severe deformity, long-term pain, and spinal cord injury. The probability of surgery should be reduced for mild and moderate adolescent patients. As such, it was of great significance to seek simple, safe, and effective nonsurgical methods [4].

For most patients, conservative treatments were advocated with respective advantages and limitations. Among them, bracing became the most commonly used conservative treatment which may reverse the progress of AIS, and it was validated in a prospective cohort study following the Scoliosis Research Society and the International Society on Scoliosis Orthopaedic and Rehabilitation Treatment criteria. In addition, the results were positively correlated with the bracing time [5, 6]. However, adolescents often had poor compliance for long-term regular treatment in clinical practice, and longer hours of bracing may generate negative effects. Mahaudens et al. [7] found that it would hinder the growth of gluteus medius in females despite enough activity duration time and oxygen uptake of muscles. In addition, it might also limit the range of motion and inhibit the development of the shoulder and hip joints. Freidel et al. [8] advocated that although with comparable clinical efficacy, there were psychological and social disorders and complications such as pressure ulcers, back pain, and decreased respiratory function, which greatly affected mental health and quality of life. Muscle deficiency appeared to be a key mechanical factor in the onset and development of AIS, and exercises were essential to strengthen muscles, especially recommended for curvatures of 30° to 40° [9]. However, an extensive hospital stay was necessary which may be unsuitable for adolescents.

As a widely used method in the world, the benefit of MT was recognized [9]. According to the International Federation of Orthopaedic Manipulative Physical Therapists (IFOMPT), the definition of MT was “Skilled hand movements intended to produce any or all of the following effects: improve tissue extensibility, increase range of motion of the joint complex, mobilize or manipulate soft tissues and joints, induce relaxation, change muscle function, modulate pain and reduce soft tissue swelling, inflammation or movement restriction” [2]. However, systematic analyses with definite conclusions of MT on AIS were lacking due to a scant number of relevant RCTs and a substantial risk of bias [10]. Thus, we conducted this meta-analysis based on extended database searches to provide better evidence of the efficacy of MT in treating AIS.

## 2. Materials and Methods

### 2.1. Criteria for including Studies

All RCTs of MT for the management of patients with AIS were included in the present study. Specifically, studies of standalone MT versus other treatments (such as MT versus exercise), MT combined with other conservative treatment versus the same conservative treatments (such as MT plus exercise versus exercise), or MT combined with other conservative treatment versus the other conservative treatments (such as MT plus exercise versus bracing plus exercise) were included. AIS was defined as a scoliotic curvature of 10° or more (measured by using the Cobb angle) including males and females between 10 and 19 years. Briefly speaking, the treatment difference between the experimental group and the control group was mainly MT or else was not included (such as MT plus exercise versus bracing). Due to the scarcity of studies, any type of RCTs that met the above criteria was included in this review. The outcomes consisted of the total effective rate, the Cobb angle, and Scoliosis Research Society-22 (SRS-22).

### 2.2. Criteria for excluding Studies

Studies of other main treatments were excluded. Non-RCTs, non-AIS, clinical experiences, trials with fewer than 10 patients, cross-sectional studies, case reports, comments, and reviews were excluded. Also, studies were excluded if they included subjects with a scoliotic curvature of less than 10° or older than 19 years.

### 2.3. Database Searches

Electronic database searches were conducted from database inception to July 2022, including the Cochrane Library, PubMed, Web of Science, Embase, Wanfang Data, China National Knowledge Infrastructure (CNKI), and Chinese Scientific Journal Database (VIP). The combinations of MeSH Terms and relevant keywords were as follows: “Manual Therapy” (MeSH Terms) OR “Manipulation” OR “Massage” OR “Chiropractic” OR “Osteopathy” OR “Acupressure” OR “Myofascial release” OR “Tuina” OR “Shiatsu” OR “mobilization” AND “Scoliosis” (MeSH Terms) OR “Adolescent Idiopathic Scoliosis” OR “Spinal Curvatures” AND “Randomized Controlled Trial” (MeSH Terms). Also, the search strategy was determined for each database. In addition, the language was restricted to English or Chinese, with no limitation on subheadings. We searched reference lists of the identified papers to explore other studies, and trials not covered in the databases mentioned above were additionally searched once identified. Duplicate studies were deleted after reviewing the abstracts and full texts. This study mainly referred to the PRISMA 12 reporting guidelines for the meta-analysis of intervention trials [11].

### 2.4. Data Collection and Analysis

The data processing was managed by two authors with EndNote X8 software independently, and disagreements were resolved by the third author. Information for each eligible study included (1) descriptive statistics such as author information, publication year and country, and data sources and sample sizes; (2) intervention characteristics such as detailed MT, concomitant treatments, and treatment course; (3) type of clinical study design and methods of randomization and blinding; (4) information of outcomes such as outcomes of interest, follow-up duration, and adverse events. We contacted authors of the included studies for additional original data if necessary. The meta-analysis was performed using RevMan 5.4 software. Statistical heterogeneity was evaluated utilizing a Chi-squared test and the *I*^2^ test. *I*^2^ value less than 25% indicated low heterogeneity and less than 50% indicated moderate heterogeneity. Then, a fixed effects model was adopted. Otherwise, an *I*^2^ value greater than 50% was regarded as significant heterogeneity and a random effects model was adopted. The standardized mean differences (SMDs) of 95% CIs were used regarding different methods of measurement. If there was significant heterogeneity, a subgroup analysis was performed.

### 2.5. Assessment of Methodological Quality

The quality of evidence was determined using the Grading of Recommendations, Assessment, Development, and Evaluation (GRADE) system for each meta-analysis [12, 13]. Factors that may decrease the quality of the evidence are the risk of bias, inconsistency, indirectness, and imprecision of outcome measures. The quality rating of the evidence started at high and was downgraded to moderate, low, or very low evidence.

## 3. Results

### 3.1. Literature Search

**Figure 1:**
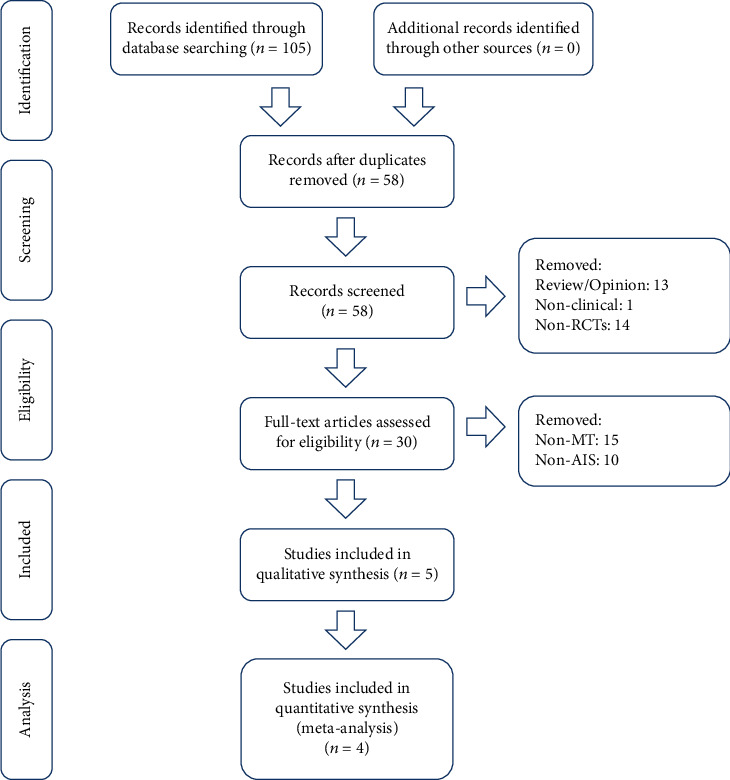
Flow diagram of the study selection process for the meta-analysis.

First, 105 studies were confirmed. Afterward, we reviewed the abstracts and titles and removed duplicates independently, resulting in 58 studies. Based on the inclusion criteria, non-RCTs, reviews, opinions, and records with inappropriate intervention approaches were excluded. Finally, 4 RCTs including 213 patients in the experimental group and 177 in the control group were included after reading the full text [14–17] (Figure 1). The characteristics of the included trials are shown in Table 1.

### 3.2. Study Characteristics

The present review collected a total of 4 RCTs published between 2019 and 2022 from China. All studies focused on the efficacy of MT in the treatment of AIS. One study was divided into 2 separate pieces of data, respectively, due to multiple groups in the study [16]. There are 2 studies of standalone MT in the experimental group [14, 16] and 3 studies of MT with the same conservative treatments in the control group [15–17].

### 3.3. Risk of Bias

**Figure 2:**
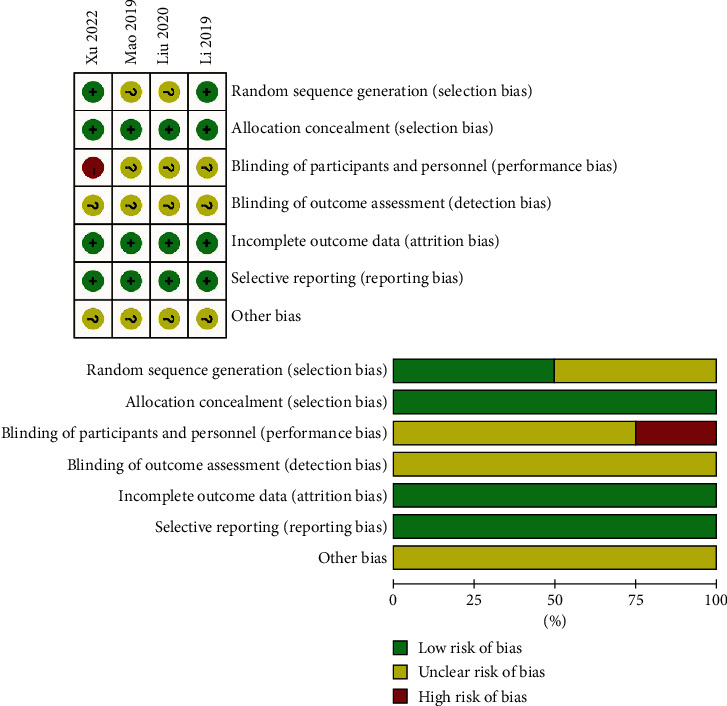
The methodological quality of the included studies. Risk of bias summary: +: low risk of bias; −: high risk of bias; ?: bias unclear.

Of the 4 included studies, all but 1 study were considered to have a low risk of bias. Random sequence generation was reported in 2 studies and allocation concealment in 4studies. Only 1 study was considered to have a high risk of bias regarding the blinding of participants and personnel, and the remaining 3 studies did not report this. Blinding of outcome assessment, incomplete outcome data, and selective reporting was not found in 4 studies (Figure 2).

### 3.4. Outcome Measures

#### 3.4.1. Total Effective Rate

**Figure 3:**
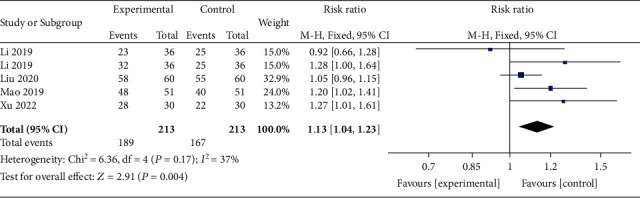
Forest plots of total effective rate after the interventions. MD = mean difference; CI = confidence interval.

Four trials including 213 subjects reported total effective rate. As shown in Figure 3, a statistically significant difference was found (*P* = 0.004), and a fixed effects model was utilized due to mild heterogeneity (*I*^2^ = 37%).

#### 3.4.2. Cobb Angle

**Figure 4:**
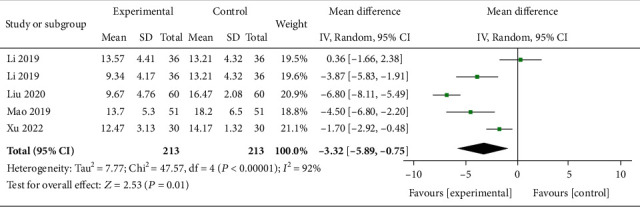
Forest plots of Cobb angle after the interventions. MD = mean difference; CI = confidence interval.

**Figure 5:**
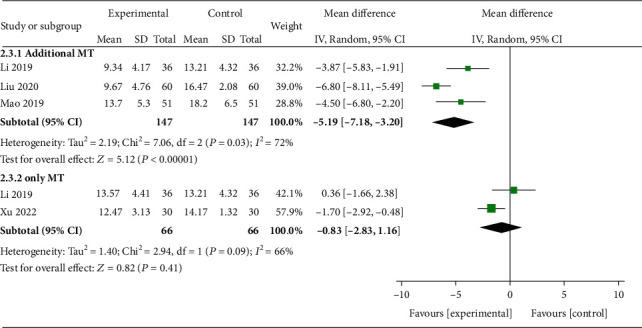
Forest plots of subgroups of Cobb angle based on additional or only MT in the experimental group. MD = mean difference; CI = confidence interval.

Four trials including 213 subjects reported Cobb angle. As shown in Figure 4, a statistically significant difference was found (*P* = 0.01), and a random effects model was utilized due to severe heterogeneity (*I*^2^ = 92%). Then, a sensitivity analysis was conducted based on additional or only MT in the experimental group as shown in Figure 5. The heterogeneity was significantly decreased. Significant differences were observed in the additional MT subgroup after interventions (*P* < 0.00001, *I*^2^ = 72%) while not in the standalone MT subgroup (*P* = 0.41, *I*^2^ = 66%).

#### 3.4.3. SRS-22 Questionnaire Scores

**Figure 6:**
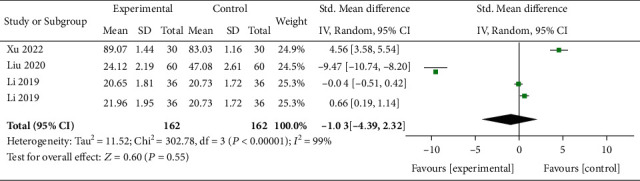
Forest plots of SRS-22 scores after the interventions. MD = mean difference; CI = confidence interval.

Three trials including 162 subjects reported SRS-22 scores. As shown in Figure 6, no statistically significant difference was found (*P* = 0.55), and a random effects model was utilized due to severe heterogeneity (*I*^2^ = 99%).

### 3.5. Sensitivity Analysis

Sensitivity analyses were conducted to evaluate the effect of individual studies on the overall outcome by sequentially removing studies. However, there was no substantial change in heterogeneity except for the Cobb angle, indicating that the above results were relatively stable.

### 3.6. Quality Assessment of Study

The strength of the evidence regarding total effective rate, Cobb angle, and SRS-22 questionnaire scores was very low due to the high risk of bias, inconsistency, imprecision, or publication bias (Table 2).

### 3.7. Publication Bias

**Figure 7:**
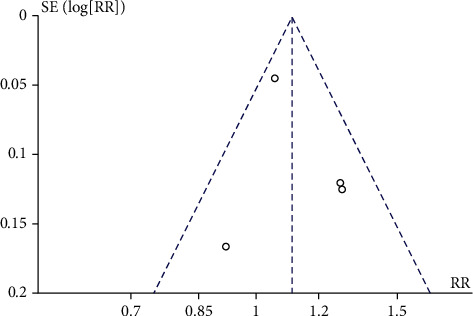
Funnel plot showing publication bias for studies comparing total effective rate between the two groups. MD: mean difference; SE: standard error.

The total effective rate was the common outcome index of 4 including RCTs, and it was also the main indicator. Therefore, the outcome index was used to make a funnel plot to detect publication bias, as shown in Figure 7. Visual inspection of the funnel plots showed symmetry, suggesting that there was no publication bias.

## 4. Discussion

The treatment of AIS is aimed at stopping curve advancing, preventing respiratory dysfunction, relieving pain, and improving the aesthetic appearance [9]. As the previous clinical trial methods were not based on the SRS criteria, MT was not a recommendation from the present evidence [18]. Also, previous systematic reviews failed to achieve any firm conclusions regarding the efficacy of MT either as a standalone or additional treatment, largely due to a limited number of RCTs [2, 10, 19]. Indeed, Chinese databases were omitted in previous studies. MT, which originated from the traditional Chinese medicine manipulation, had long been applied to the clinical diagnosis and treatment, which was recorded in *The Yellow Emperor's Classic of Internal Medicine*, the earliest medical classic now extant and written about 2500 years ago. Moreover, innovations were developed in modern MT by incorporating local anatomy and biomechanical principles, to adjust the abnormal spinal position and release the soft tissue surrounding the spine, especially on the concave side. Thus, we aimed to provide better evidence of the efficacy of MT for AIS.

In theory, MT was divided into two types: chiropractic technology for spinal alignment and manipulation for myofascial release. Pressure and touch were thought to take effect by restoring lymphatic drainage, improving blood circulation, lengthening short or tight connective tissue, relaxing tense muscles, and soothing the nervous system [20]. In the application of chiropractic techniques, the first step was to identify the responsible segments. Through the rapid force on the joint, it moved into the physiological range more than the elastic range, to correct the spinal position [21]. Based on this, chiropractic was thought to correct spinal distortion, restore muscle imbalance, even restore the function of spinal nerves [22], and stimulate the Golgi tendon organ located around the tendon to relax the muscle and expand its range of motion [23].

In the present study, it was still currently insufficient to confirm the validity of spinal manipulation due to the very low quality of included studies, although there were positive results. Ideally, one of the assessments of bracing included the percentage of patients who have ≤5°curve progression per year, at skeletal maturity and two years after ending the bracing, and the percentage of patients who have >5° progression up to skeletal maturity [24, 25]. We referred to the loss of progression (>5°) as the total effective rate that was the most often used in the included literature. A statistically significant difference was found in the total effective rate (*P* = 0.004). Also, a statistically significant difference was found in the Cobb angle (*P* = 0.01), especially in the additional MT subgroup (*P* < 0.00001). However, the fact that the longest follow-up was 2 months in the included studies did not support any firm conclusions. Indeed, a minimum of 12 months was needed for nonoperative research according to the SOSORT and SRS guidelines [26]. In 2 studies included, MT was administered along with electric acupuncture and traction. The results of the included studies suggested that additional spinal manipulative therapy may be promising for the management of AIS. After a session or a period of manual treatment, it was very difficult to maintain the corrective effect like bracing. Thus, very short-term results were considered only for bracing [27]. Moreover, the effect of acupuncture in the treatment of patients with scoliosis was not determined [28]. Based on the above, the short-term effectiveness of spinal manipulation as an adjunct therapy was either not confirmed. Evaluation of skeletal maturity is the key factor in determining the treatment strategy. However, as the most commonly used maturity indicator, the Risser sign was omitted from included studies. In general, patients older than 16 years had little growth activity and a risk of progression [29]. Considering the wide range of patients' ages in the included study, there was still lacking high-quality evidence for the radiological outcome.

The main advantage of SRS criteria was to focus the study on patients at high risk of progressing to the surgical level. Strict inclusion criteria suppressed research efforts for nonsurgical treatment of scoliosis in other patients. Thus, the SOSORT and SRS guidelines highlighted the significance of clinical outcomes relevant to patients such as aesthetics, disability, pain, and quality of life [26]. In this study, 3 trials reported SRS-22 scores (*P* = 0.55) without significant differences, and the strength of the evidence was very low. Similarly, a previous meta-analysis also stated that there was uncertainty about the effect of segmental spinal mobilizations on increasing quality of life in AIS due to very low-quality evidence (serious risk of bias, unknown inconsistency, and very serious imprecision) [30]. Indeed, the effect on improving the quality of life had not been observed, and it may be related to the concealment of symptoms in AIS. Overall, this requires further RCTs with rational follow-up times to fully evaluate. In retrospect, Theroux et al. [10] failed to establish evidence as only 4 studies with lack of controls were included, which could not be quantitatively analyzed. By expanding the scope of the database and focusing on specific manual techniques, Driehuis et al. [30] included more studies. The included very low-quality studies still made it difficult to draw definitive conclusions. Nevertheless, he noted the importance of intermediate outcomes, detailed description of the technique, and RCT designs. We further expanded the search scope to include the RCTs. Although quantitative analysis was carried out, no definitive conclusions could be drawn limited by very low study quality. It is recommended that MT (gentle, short-term mobilization, or releasing soft tissues techniques) was proposed only if associated with stabilization physiotherapeutic scoliosis-specific exercises [18]. Given the above results, we suggested that future studies focus on exploring the short-term effects as an adjuvant therapy and evaluating clinical outcomes relevant to patients.

The lacking identification of underlying pathogenesis was the main reason for hindering clinical progress. Considering that AIS is fundamentally a structural malformation, at least we could have an explanation in terms of mechanics or the forces involved. In recent years, myofascial chain theory was developing and formed a complete system [31]. The dorsal myofascial chain was the dominant chain in the sagittal plane for the coordination of position and action. When spinal scoliosis occurred, dorsal asymmetry led to imbalance and symptoms. Physicians could adjust the muscle tension, restore the overall balance, and correct the position of the spine through the integral analysis of the myofascial chain state. In addition, the etiology of AIS may be closely associated with increased musculoligamentary tension [32]. Through a thoracolumbar physical model, Crijns et al. demonstrated that primary differential tendon elongation in the sagittal plane resulted in internal compression of the spine and the subsequent inevitable lateral bending and axial rotation [33]. Intervertebral disc height in AIS patients was relatively larger than normal [34, 35] which was strongly correlated with low muscle strength [36, 37] and reduced spinal axial loading. As such, the longitudinal ligament with insufficient ligament adaptation to mechanical stretch may stop remodeling and growing [38], resulting in a scoliotic curve and rotation. Moreover, it may also trigger a cytokine-mediated cascade toward tissue repair [39], resulting in scar tissue formation in the ligaments [40].

### 4.1. Limitations

The study had the following limitations. First, all studies did not meet the inclusion criteria based on the SOSORT and SRS guidelines. In particular, the included studies did not have sufficiently long follow-up periods. According to the consensus recommendations, the following periods included: short term (at least 12 months of treatment), end of bone growth (Risser+3/4), end of treatment (at treatment discontinuation), and final results at full-bone maturity. In light of the short-term result, the effectiveness as an adjunctive therapy of bracing can be evaluated in the future. It is of great importance to encourage high-quality, principled research in compliance with the guidelines. Second, there was a lack of standard manipulative procedures and unified quantitative evaluation which may increase heterogeneity and prevent the translation of study findings to clinical practice [30]. Third, to fully evaluate the benefits and risks, detailed side effects should be considered and documented in clinical trials of any treatment [41]. Based on the above, more rigorous RCTs are needed in the future to determine efficacy and facilitate standardized treatment regimens.

## 5. Conclusions

Based on the GRADE methodology, the evidence was of very low quality. There is insufficient data to determine the effectiveness of MT. High-quality studies with appropriate design and follow-up period are warranted to determine if MT may be beneficial as an adjunct therapy for AIS. Currently, there is no evidence to support spinal manipulation.

## Figures and Tables

**Table 1:** Characteristics of all the trials included in the meta-analysis.

Study	Year	Country	Sample size		Age (years)		Cobb angle (°)		Interventions	
			E	C	E	C	E	C	E	C
Zhihao [17]	2019	China	51	51	15.7 ± 1.8		23.5 ± 6.8	23.8 ± 6.5	MT+traction	Traction
Rui and Jian [14]	2022	China	30	30	12.37 ± 1.25	12.57 ± 1.31	21.70 ± 2.26	21.30 ± 1.93	MT	Schroth therapy
Zhiyong et al. [16]	2019	China	36^a^	36	13.34 ± 0.77	13.52 ± 0.71	20.64 ± 4.67	20.17 ± 4.72	MT+electric acupuncture	Electric acupuncture
			36^b^	36	13.46 ± 0.89	13.52 ± 0.71	20.31 ± 4.58	20.17 ± 4.72	MT	Electric acupuncture
Jia et al. [15]	2020	China	60	60	14.57 ± 2.59	14.62 ± 2.92	19.26 ± 2.14	19.39 ± 2.69	MT+traction+excercise	Traction+excercise

E: experimental group; C: control group; MT: manual therapy; ND: the study did not report this information. ^a,b^Patients were analyzed separately due to multiple treatments.

**Table 2:** Meta-analyses of effect of MT.

Outcomes	Number of studies	Number of subjects	Effect	*I* ^2^	Quality of evidence (GRADE)
Total effective rate	4	213	RR 1.13 (1.04 to 1.23)	37%	Very low^∗^^†#^
Cobb angle	4	213	MD 3.32 lower (5.89 to 0.75 lower)	92%	Very low^∗^^††‡#^
Subgroups (additional MT)	3	147	MD 5.19 lower (7.18 to 3.20 lower)	72%	Very low^∗^^††^
Subgroups (only MT)	2	66	MD 0.83 lower (2.83 lower to 1.16 higher)	66%	Very low^∗^^†‡#^
SRS-22 questionnaire scores	3	162	SMD 1.03 lower (4.39 lower to 2.32 higher)	99%	Very low^∗^^††‡#^

GRADE, GRADE working group grades of evidence. ^∗^Risk of bias results downgrade. ^†^Inconsistency results downgrade. ^‡^Imprecision results downgrade. ^#^Publication bias downgrade.

## Data Availability

The datasets used and/or analyzed during the present study are available from the corresponding author upon reasonable request.
